# Using Wearable Inertial Sensors to Estimate Clinical Scores of Upper Limb Movement Quality in Stroke

**DOI:** 10.3389/fphys.2022.877563

**Published:** 2022-05-03

**Authors:** Charlotte Werner, Josef G. Schönhammer, Marianne K. Steitz, Olivier Lambercy, Andreas R. Luft, László Demkó, Chris Awai Easthope

**Affiliations:** ^1^ Spinal Cord Injury Research Center, University Hospital Balgrist, Zurich, Switzerland; ^2^ Rehabilitation Engineering Laboratory, Department of Health Sciences and Technology, ETH Zurich, Zurich, Switzerland; ^3^ Cereneo Foundation, Center for Interdisciplinary Research (CEFIR), Vitznau, Switzerland; ^4^ Division of Vascular Neurology and Neurorehabilitation, Department of Neurology and Clinical Neuroscience Center, University of Zurich and University Hospital Zurich, Zurich, Switzerland; ^5^ Future Health Technologies, Singapore-ETH Centre, Campus for Research Excellence and Technological Enterprise (CREATE), Zurich, Singapore; ^6^ Cereneo, Center for Neurology and Rehabilitation, Vitznau, Switzerland

**Keywords:** inertial sensor, rehabilitation, wearables, clinical assessment, stroke, ARAT

## Abstract

Neurorehabilitation is progressively shifting from purely in-clinic treatment to therapy that is provided in both clinical and home-based settings. This transition generates a pressing need for assessments that can be performed across the entire continuum of care, a need that might be accommodated by application of wearable sensors. A first step toward ubiquitous assessments is to augment validated and well-understood standard clinical tests. This route has been pursued for the assessment of motor functioning, which in clinical research and practice is observation-based and requires specially trained personnel. In our study, 21 patients performed movement tasks of the Action Research Arm Test (ARAT), one of the most widely used clinical tests of upper limb motor functioning, while trained evaluators scored each task on pre-defined criteria. We collected data with just two wrist-worn inertial sensors to guarantee applicability across the continuum of care and used machine learning algorithms to estimate the ARAT task scores from sensor-derived features. Tasks scores were classified with approximately 80% accuracy. Linear regression between summed clinical task scores (across all tasks per patient) and estimates of sum task scores yielded a good fit (*R* ^2^ = 0.93; range reported in previous studies: 0.61–0.97). Estimates of the sum scores showed a mean absolute error of 2.9 points, 5.1% of the total score, which is smaller than the minimally detectable change and minimally clinically important difference of the ARAT when rated by a trained evaluator. We conclude that it is feasible to obtain accurate estimates of ARAT scores with just two wrist worn sensors. The approach enables administration of the ARAT in an objective, minimally supervised or remote fashion and provides the basis for a widespread use of wearable sensors in neurorehabilitation.

## 1 Introduction

Neurological health conditions, such as stroke (Lindsay et al., 2019), traumatic brain injury (Dewan et al., 2019), multiple sclerosis, spinal cord injury, and Parkinson’s disease (Feigin et al., 2017) are major causes of disability, often leading to limitations in motor functioning of the upper limbs (Katz et al., 1998; Broeks et al., 1999; Hendricks et al., 2002; Kwakkel et al., 2003; Kister et al., 2013). In accordance with the International Classification of Functioning, Disability, and Health (ICF), motor functioning is typically analyzed at different levels of granularity, at the level of body joints and segments (ICF function level) and at the level of the execution of movement tasks (ICF activity level) (World Health Organization, 2001). The ICF further distinguishes motor functioning observed in controlled settings and in the person’s natural/home environment (ICF capacity and performance). The measurement of motor functioning is a vital part of both research and practice in neurorehabilitation as it provides the basis for the evaluation of new rehabilitation programs (Brunner et al., 2017), new medications (Samuel et al., 2017), prediction of recovery (Wolf et al., 2021) as well as the design of patient-specific interventions.

The current gold standards for the measurement of motor functioning are mainly based on standardized clinical tests (Kwakkel et al., 2017; Pohl et al., 2020; Prange-Lasonder et al., 2021), in which patients perform a series of pre-defined movements in standardized conditions and experts score each movement on pre-defined criteria, such as task completion, task duration and kinematic and kinetic characteristics (Demers and Levin, 2017). The tests must satisfy specific requirements in terms of both psychometric properties (validity, reliability, responsiveness) (Murphy et al., 2015) and clinical applicability (time and ease of training, administration, scoring, interpretation, cost) (Prange-Lasonder et al., 2021).

An emerging requirement regarding clinical applicability is that the tests should be suitable for the entire rehabilitation process from in-clinic to ambulant and home settings (further referred to as continuum of care). This is desirable since neurorehabilitation is expected to shift to patients’ homes due to capacity limitations in healthcare and advances in home-based rehabilitation technologies (Lambercy et al., 2021). However, the need for a trained evaluator to conduct a clinical test conflicts with the goal of ubiquitous measurement protocols.

Another requirement is that assessments should take into account movement quality (Kwakkel et al., 2017). Movement quality refers to the degree to which patients’ motor execution of a task resembles that of normal individuals (Kwakkel et al., 2019). High movement quality is associated to the restitution of pre-morbid movement execution patterns, whereas low movement quality is linked to alternative (compensatory) movement patterns (Demers and Levin, 2017; Jones, 2017). Specifically, task execution of patients with neurological disorders is typically characterized by slow and jerky movements of the arm end point, abnormal grasping, reduced elbow extension, and increased shoulder abduction compared to age-matched healthy individuals (Saes et al., 2022).

Ideally, movement quality should be quantified with kinematic measures (Saes et al., 2022). However, the identification of kinematic measures of arm movement quality is challenging because many kinematic parameters exist (Schwarz et al., 2019), their relevance depends on the specific movement task (Schwarz et al., 2019), selected kinematic measures require extensive psychometric validation (Murphy et al., 2011; Alt Murphy et al., 2012; Thrane et al., 2020; Frykberg et al., 2021), and the measurement systems are usually stationary, expensive, and require expert users (Alt Murphy et al., 2018).

Due to the difficulties with establishing kinematic measures of movement quality studies started to explore an intermediate goal. Supervised machine learning algorithms and low-cost sensor data were used to estimate clinical test scores (for reviews see Oña Simbaña et al., 2019; Kim et al., 2021; Boukhennoufa et al., 2022). This approach has the advantages that the clinical tests have established psychometric properties (Kim et al., 2021), that clinical scores are easy to interpret (Kim et al., 2021) and that wearable movement sensors can be used which are low cost and enable data collection across the entire continuum of care (Oña Simbaña et al., 2019; Kim et al., 2021; Boukhennoufa et al., 2022). Tests of ICF activity capacity assess limitations in the accomplishment of tasks that are relevant for activities of daily living (Prange-Lasonder et al., 2021). Importantly, clinical scores of ICF activity capacity often contain information about movement quality since evaluators visually examine movement quality to determine the test scores (Yozbatiran et al., 2008; Sapienza et al., 2017; Adans-Dester et al., 2020).

One of the most prominent clinical test of upper-limb ICF activity capacity is the ARAT (Lyle, 1981; Yozbatiran et al., 2008), which provides a combined score comprising the aspects of movement speed, successful task completion, and hand and arm movement quality (Yozbatiran et al., 2008). In the ARAT, a patient performs several tasks that require combined reaching and grasping. Performance in each task is rated on an ordinal scale depending on task duration and observed movement quality characteristics (e.g., smoothness of the arm endpoint, abnormal grasp, compensatory movements) (Lyle, 1981; Yozbatiran et al., 2008). Individual task scores are then summed up to a total score (Lyle, 1981; Yozbatiran et al., 2008). The ARAT is the most frequently used assessment of upper-limb functioning in clinical studies (Murphy et al., 2015), as it is used in a broad range of neurological health conditions such as stroke, traumatic brain injury, multiple sclerosis (Prange-Lasonder et al., 2021) and Parkinson’s Disease (Song, 2012), has excellent psychometric properties (Pike et al., 2018), is widely accepted and recommended by experts (Kwakkel et al., 2017; Pohl et al., 2020; Prange-Lasonder et al., 2021), and is a significant predictor of motor recovery in stroke (Wolf et al., 2021). Despite the importance of the ARAT, however, wearable sensor data were never utilized to estimate the test outcome, to the best of our knowledge (Oña Simbaña et al., 2019; Kim et al., 2021; Boukhennoufa et al., 2022).

In the current study, we collected data of stroke patients performing the ARAT while two inertial sensors were attached to their wrists. ARAT task and total scores were estimated using supervised machine learning. We hypothesize that with this approach it is feasible to estimate ARAT scores with an error that is similar or smaller than clinically relevant changes, namely, the minimally detectable change (Simpson and Eng, 2013) and the minimal clinically important difference of the ARAT task and total scores (Van Der Lee et al., 2001). Such sensor-based estimates of clinical scores may pave the way for automated, expert-independent administration. In addition, the simple setup of using just two wearable sensors enables location independent measurements with the potential to be used across the whole continuum of care.

## 2 Methods

The current study was a secondary analysis of data collected under a randomized-controlled trial (Steitz et al., 2022; Kantonale Ethikkommission Zentralschweiz, approval number: BASEC:2017-00199) and during an evaluation of sensor types in clinical routine (Kantonale Ethikkommsion Zentralschweiz, request number: Req-2020-00995). Both studies adhered to the Declaration of Helsinki. Participants were recruited at the University Hospital Zurich and the Center for Neurology and Rehabilitation cereneo, and gave informed consent prior to both studies.

### 2.1 Participants

Participants were included if they were 1) 18 years of age or older, 2) in a sub-acute stage of stroke (3–90 days after symptom onset) with lateral ischemia (or hemorrhage) as confirmed by brain imaging and 3) showed subsequent impairment of arm function with a Fugl-Meyer Assessment for the Upper Extremities (FMA-UE) score between 15 and 59 points. Participants were excluded in case of 1) other neurological disorders that might result in dementia, cognitive dysfunction or central motor symptoms, 2) severe sensory aphasia, 3) preexisting arm paresis, 4) intake of sedatives or neuroleptics, or 5) relevant hearing.

Data of 21 participants who satisfied these criteria were acquired. The age of the participants was 68 ± 10 years (mean+/-standard deviation), out of which 5 were female and 20 right-handed. All patients were in a subacute stroke stage at the time of the first assessment, with symptom onset 38 ± 17 days before the assessment. All patients had lateralized ischemia or hemorrhage as confirmed by brain imaging, and suffered subsequent impairment of the arm function, i.e., the FMA-UE score was 33 ± 15 points. The median of the total clinical ARAT score of the 21 patients was 35.5 (interquartile-range: 19.5–47.3) and 57 (interquartile-range: 45–57) for the more and less affected sides, respectively. The study population thus covered a broad range of patients with different upper extremity motor function.

### 2.2 Apparatus, Instruments, and Procedures

**FIGURE 1:**
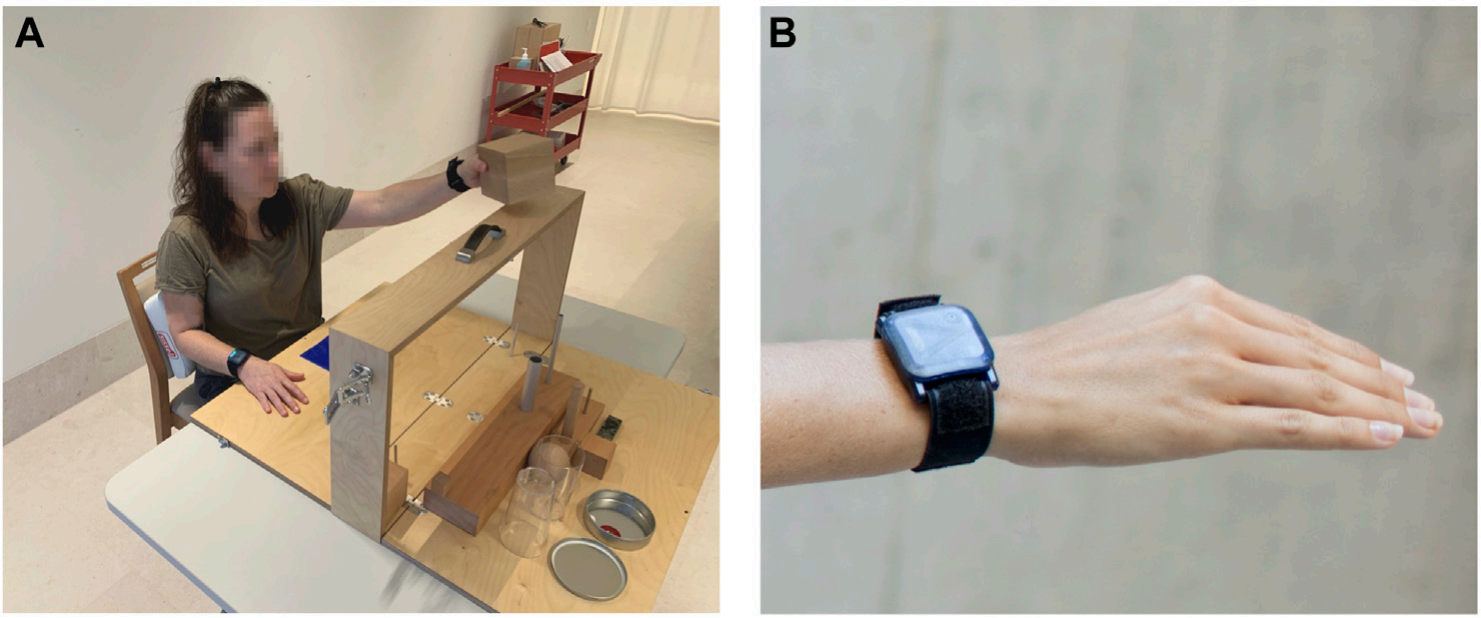
**(A)** A participant performing ARAT task 1, which is part of the grasp domain. The task is to grasp a wooden block (10 cm in size) at the start position (blue patch on the table) and to put it on the shelf in front of the subject. **(B)** Close-up of the inertial sensor attached to the wrist.

The ARAT was administered twice per participant, at baseline and 1–4 weeks later. The ARAT comprises of 19 movement tasks that are grouped into four domains (grasp, grip, pinch and gross movements). Each task is performed with the less impaired arm first and the more impaired arm second, and assigned an ordinal rating with a range from 0 to 3. The ARAT was conducted with standardized materials (Figure 1A) and procedure (Yozbatiran et al., 2008), with one exception: In the standard procedure, subjects make an attempt on the first, most difficult task in each domain, and, in case of normal functioning, skip the remaining, easier tasks of the domain. In this study, however, all task were administered to maximize the data obtained from each participant. The performance of each movement of each patient has been assessed by one of two experienced evaluators, resulting in a maximum achievable total score of 57 per arm and per test. Since the ARAT is highly standardized and has high inter-rater and test-retest reliabilities (ICC < 0.98) (Van Der Lee et al., 2001), we believe that the selection of the evaluators does not affect the rating results.

During the assessment, wearable inertial sensors (ZurichMOVE, Switzerland) were tightly attached to each wrist with custom-made flexible straps as shown in Figure 1B. The main components of the sensor modules are a tri-axis accelerometer, gyroscope and magnetometer, measuring at a sampling frequency of 50Hz, which is sufficient given that there was no aim to reconstruct the actual movement trajectories. The magnetometer data was excluded from the analysis, because magnetic fields are often distorted indoors, and thus the magnetometer data is considered to be unreliable. Furthermore, the timestamp of the beginning and end of each task was recorded.

### 2.3 Data Preprocessing and Analysis

Since accelerometers measure both the movement acceleration and gravity, the gravitational component has to be subtracted from the acceleration signal. For this, the orientation of the sensors in space was obtained by using the sensor fusion algorithm developed by Seel and Ruppin (2017). This algorithm is based on an analytical solution to remove the drift in the inclination angle with the information of the direction of gravity from the accelerometer. Based on the sensor orientation the acceleration data could be transformed from the moving coordinate system into a coordinate system fixed in space. In this fixed coordinate system, the gravitational component is pointing in the vertical direction and can thus be easily removed by subtracting g from this axis. This procedure resulted in the pure movement acceleration data.

The tri-axial acceleration and angular velocity data was then segmented according to the recorded start and stop times of each task. This resulted in 6D time series sequences of different lengths, depending on how long the patient needed to perform the given task. Short sequences lasted around 1–2s, while the maximum sequence length was limited to 60s (as per ARAT definition if the patient was unable to complete the task within this time). In rare cases ( < 6.7%), data were missing due to technical problems or because the patient did not attempt to perform the task. In such cases, the patient received a score of 0 for this task, and a sequence of non-moving data of 10s from this patient was used in order to have complete data sets.

### 2.4 Feature Extraction and Classification

The machine learning approach used in this study required features for the classification. Hence, descriptive features were extracted from each time series sequence. The selection of features was based on the recommendations of Suto et al. (2017) for human activity recognition. In order to characterize the sequences of each task in the time domain, the following features were computed for each axis of the acceleration and angular velocity time series data: mean, standard deviation, minimum (defined as the 5th percentile), maximum (95th percentile), range (minimum to maximum), mean absolute deviation, interquartile range (25th to 75th percentile), upper quartile (75th percentile), zero-crossing rate, and kurtosis. To characterize the frequency spectrum of the data, a fast Fourier transform was applied to the vector-wise norm of acceleration and angular velocity time series data of each task. The following features were extracted: maximum frequency component, spectral energy of different frequency ranges (0–5 Hz, 5–10 Hz, 10–15 Hz, 15–20 Hz, and 20–25 Hz), and spectral centroid. This resulted in altogether 74 features for each task: 60 features characterizing the movement in the time domain and 14 features characterizing the movement in the frequency domain.

The model received these sensor-derived features as an input to estimate the 4-point scale ARAT task scores. All features were standardized by centering them around the mean and scaling them to have unit variance in order to provide features of similar magnitude to the classifier. An ordinal classifier as described by Frank et al. (Frank and Hall, 2001) was chosen as a model to consider the ordinal ranking of the four ARAT task scores. A logistic regression was then selected as classifier, and regularization was used to prevent overfitting on the training data. This ordinal logistic regression classifier was trained individually for each of the four ARAT domains, because the movements within these domains differed significantly. The grasp and pinch domains consist of pick-and-place tasks that differ in terms of grasping type. Tasks of the grip domain on the other hand resembles daily life activities, e.g., pouring water from a bottle to a glass, while the gross domain includes shoulder and arm movements across a wide workspace. The separation into the four domains fostered each classifier to differentiate between different executions of the same movement task as opposed to training a single classifier on all tasks, which would have needed to handle the high variability introduced by the different nature of the movement tasks.

**FIGURE 2:**
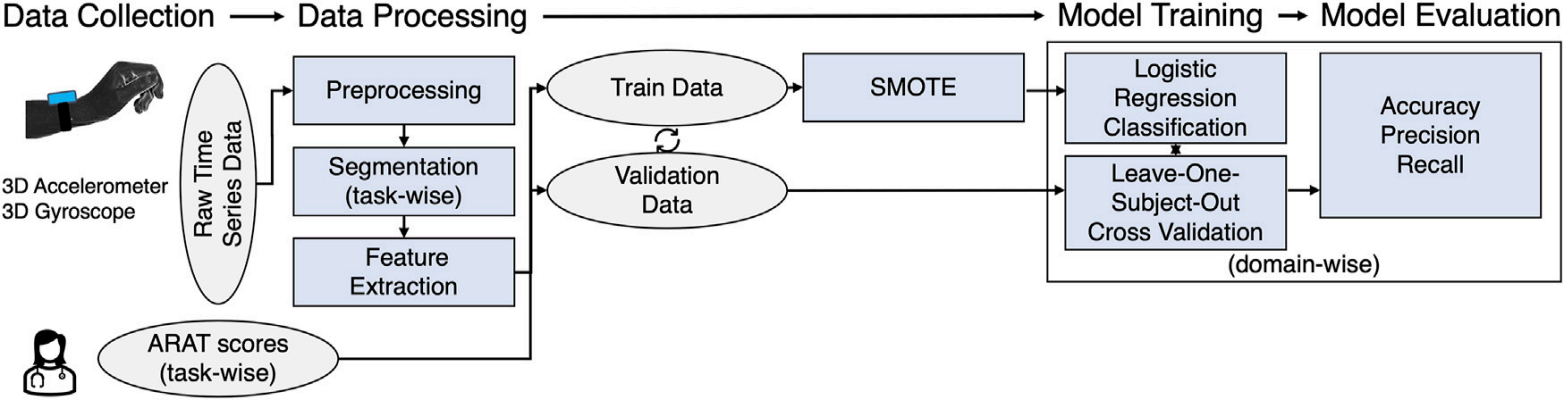
Flow chart of the framework to estimate task-wise ARAT scores from inertial sensors attached to the wrist.

The less affected arm achieved the maximal score in many of the subtasks, which resulted in a highly unbalanced data set. To counteract this, the training data set has been balanced by upsampling the number of rare observations using the synthetic minority over-sampling technique (SMOTE) (Chawla et al., 2002). Due to the small sample size leave-one-subject-out cross-validation procedure was used to test the classifiers on unknown data. More specifically, the upsampled data of all subjects and all sessions except for the data of one subject and both sessions (if available) was used to train the model, which was then tested on the original (non upsampled) data of the remaining subject. This process was repeated until the model was tested on the data of all subjects. A flowchart of the data processing and classification workflow is displayed in Figure 2.

### 2.5 Evaluation of the Model

The performance of the classifiers for each domain has been estimated based on accuracy, precision, and recall computed from the clinically assessed (further referred to as clinical) ARAT task scores and the estimated ARAT task scores. These metrics were weighted by the distribution of the samples within the classes to account for class imbalances. For each arm, the estimated task-level ARAT scores were summed up to yield an estimate of the total ARAT score. Linear regression was used to study the relationship between the clinical and the estimated total ARAT scores. Furthermore, the mean error and the root mean squared error (RMSE) were computed as the average and the root mean squared of the differences between the estimated and the clinical ARAT scores, respectively.

## 3 Results

### 3.1 Estimation of the Action Research Arm Test Task Scores

**FIGURE 3:**
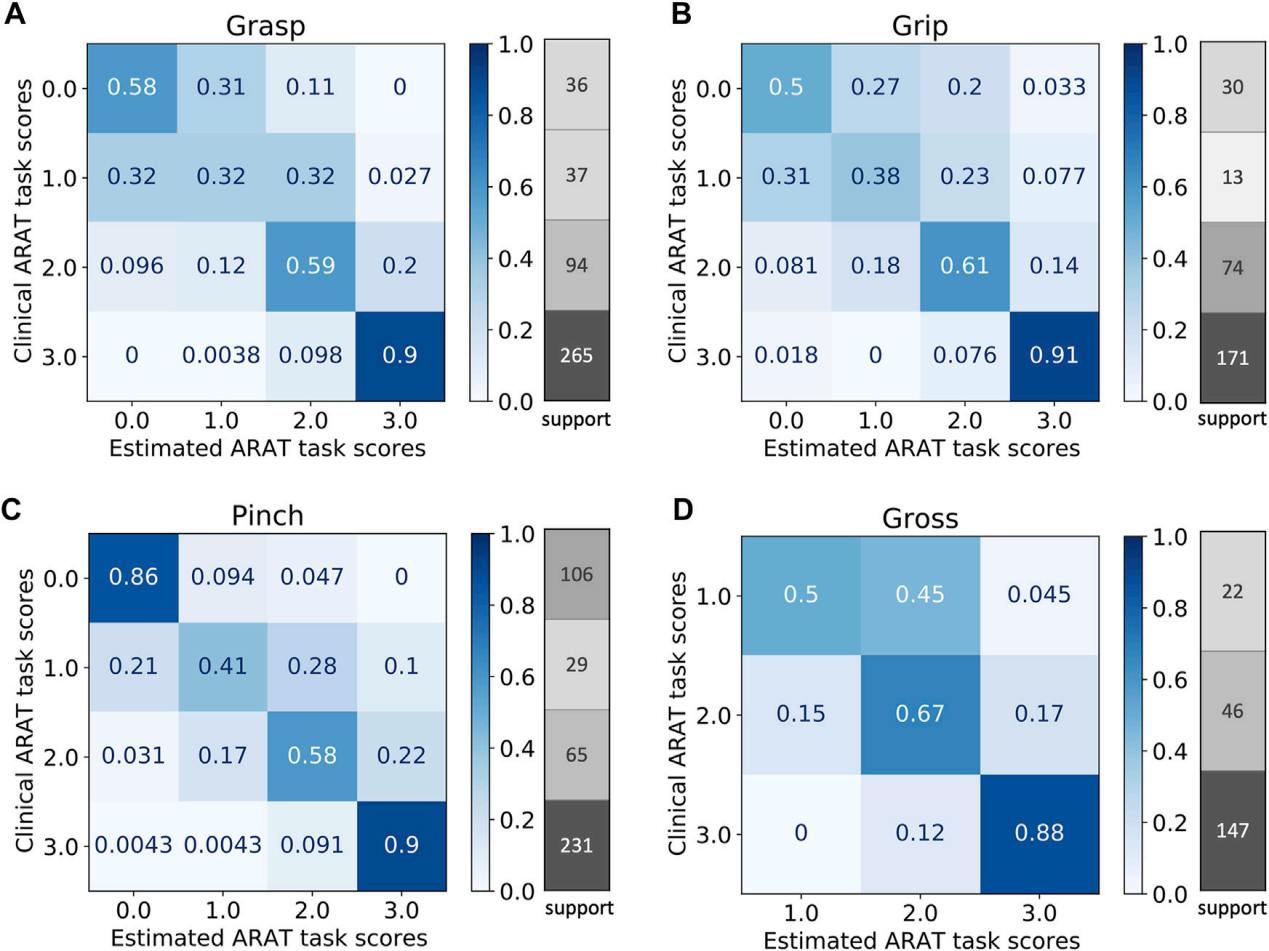
Normalized confusion matrices and number of observations per class (support) for the four domains: grasp **(A)**, grip **(B)**, pinch **(C)**, and gross **(D)**.

The measurement of the 21 patients resulted in 1,366 observations altogether (2 observations had to be exuded) that were divided into the four domains to train the ordinal classifiers. No patient received a score 0 in any of the tasks of the gross domain. Hence, the gross classifier was only trained on three classes. For all domains, the classifiers identified the task scores of three well. However, the classifiers had difficulties discriminating score 1 from 0 to 2, which were also the cases with fewer number of observations in comparison to the other cases. The normalized confusion metrics and number of observations per class are shown in Figure 3.

The four ordinal classifiers estimated the ARAT task scores from the sensor-based features with a weighted accuracy ranging from 76% (grasp) to 81% (pinch) as evaluated by the leave-one-subject-out cross-validation and summarized in Table 1. For the pinch and gross domains, weighted accuracy, precision, and recall values of above 0.8 were obtained. The classifiers performed slightly worse for the grasp and grip domains, where values below 0.8 were obtained for accuracy, precision, and recall. Note that the unbalanced nature of the data affects the weighted accuracies. More specifically, score 3, which was classified with high accuracy, has a strong influence on the overall accuracy as it was the most frequent observation, while the other, more infrequent scores, which were classified with low accuracy, have less impact.

**TABLE 1:** Overview of performance of the model predicting the ARAT task scores in the four domains: weighted accuracy, precision, and recall.

Domain	Accuracy	Precision	Recall
Grasp	0.75	0.76	0.75
Grip	0.76	0.79	0.76
Pinch	0.81	0.82	0.81
Gross	0.80	0.81	0.80

### 3.2 Estimation of the Total Action Research Arm Test Score

**FIGURE 4:**
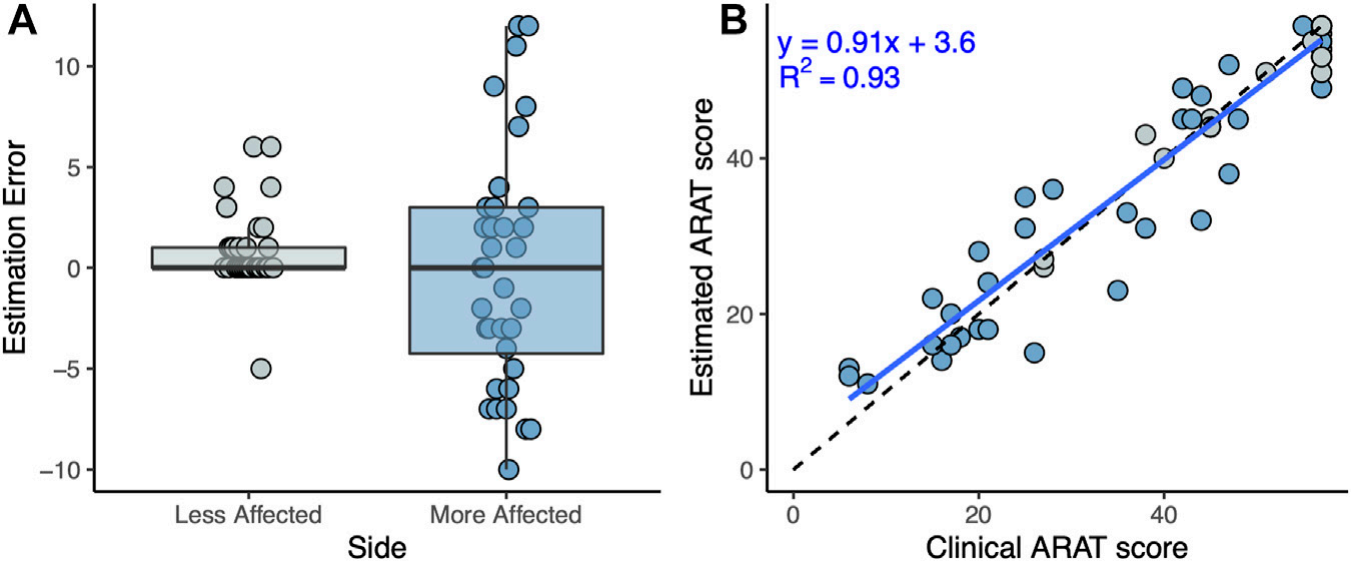
**(A)** Box plots showing the estimation error of the total ARAT scores for the more and less affected sides. **(B)** Linear regression (blue line) between the clinical and estimated total ARAT scores. The dashed line depicts the ideal case of y = x.

The total ARAT score, obtained by a summation of the estimated ARAT tasks scores, showed a mean error of 0.5, a mean absolute error of 2.9 points with a maximal error of 12 points. A RMSE of 4.7 was obtained. Relative to the maximum achievable total score of 57, this is a relative error of 8.2%. Higher estimation errors were obtained for the more affected side in comparison to the less affected side as depicted in Figure 4A. A linear regression between the clinical and estimated total ARAT scores resulted in a good fit (*R* ^2^ = 0.93) as plotted in Figure 4B, close to the ideal curve (y = x).

## 4 Discussion

The objective of this work was to determine whether a simple and fast setup of wearable sensors is sufficient to estimate clinical ARAT scores given by a trained evaluator. Successful estimation of ARAT is a first step toward evaluator-free measurement of ICF activity capacity and upper limb movement quality. For this purpose, data of 21 patients performing the standardized ARAT assessment while wearing two wrist-worn inertial sensors was recorded. By applying machine learning techniques to the time series sensor data, ARAT scores could be estimated at the task level. More specifically, ordinal classifiers were trained on the balanced observations of each domain, and the performance of the classifiers was evaluated by cross-validation using typical machine learning metrics. In addition, the estimated total score, which was obtained by the summation of all the task scores, was compared to the clinical total score.

Overall, the weighted averages of the classification accuracies of the task scores were around 80% for all ARAT domains, ranging from 32 to 91% for the individual classes within a domain. Differences in performance of the classifiers might have several reasons. First, the domains differ in the homogeneity of the movements within the respective sets of tasks. In particular, the tasks in the grip domain require relatively dissimilar movements and, hence, the classifier had more difficulties to distinguish between different movement qualities. For example, one task is to pour water from one glass to another, whereas another task is to grasp a washer, to transfer it forward and to put it over a bolt. In contrast, the tasks in the pinch domain afford relatively similar movements, as all tasks consist of pick-and-place actions, where the main difference only lies in the tested fingers. Consequently, better classification performance was observed for the pinch domain. Second, the distributions of observations across the classes (i.e., the test scores) showed different degrees of imbalance between the domains. For example, the distribution of observations in the pinch domain was relatively well balanced and, accordingly, relatively high classification performance was observed. Conversely, in the remaining domains the distribution of observations was skewed even more toward higher test scores. This issue was addressed with the SMOTE oversampling technique. But certainly synthetically generated observations cannot substitute actual observations and, consequently, we observed lower classification performance in these domains. Third, in the gross movement domain we did not obtain any observation of score 0. Hence, for this domain the classifier only needed to be trained on three classes, which explains the rather good performance of this classifier compared to the classifiers of the other domains. Furthermore, classification accuracies differed between the task score levels. Specifically, for the grasp, grip, and pinch domain, the classifiers had difficulties to discriminate failure to complete the task even partially (score 0) from a partial completion (score 1), and a partial completion (score 1) from a completion of the task with great difficulty (score 2). An explanation might be that the extracted features captured this information only partially. The results suggest that the differences in wrist movements for these scores are minimal, and additional sensors, e.g., attached to the hand, could be beneficial to better identify the completeness of the task. In addition, high inter-subject variability in execution of the tasks (probably due to the different sensorimotor impairments of this patient population) and few observations of score 1 might have prevented more accurate identification of this score.

Estimates of the total ARAT score showed a mean absolute error of 2.9 points of the estimated total ARAT score as compared to the clinical total score. This error is below the minimally detectable change (MDC: 3.5 points) (Simpson and Eng, 2013) achieved by trained observers and our maximal error of 12 points is also below the minimal clinically important difference of ARAT found in the literature (MCID: 12-17 points) (Van Der Lee et al., 2001). The good fit of the linear regression between the estimated and clinical ARAT total scores (*R* ^2^ = 0.93) suggests that our approach is suitable to generate accurate estimates of the ARAT total scores. Consequently, our method has an accuracy of clinical relevance and is precise enough to detect clinically important changes in the ARAT. These good results at the sum score level suggest that errors on the tasks level might have averaged out.

Using only wrist worn sensors, one might expect inferior results, as wrist worn sensors neither directly measure movements of the elbow joint or trunk which are highly correlated with the ARAT scores (Alt Murphy et al., 2012), nor do they capture finger and hand movements which are visually examined by experts when rating the ARAT performance. However, wrist worn sensors directly capture wrist motion which is linked to movement quality aspects such as the speed and smoothness of arm movements (Kwakkel et al., 2019). These kinematic variables are known to be correlated with the ARAT scores (Carpinella et al., 2014; Repnik et al., 2018), which explains the fact that we nevertheless achieved good classification results. Additionally, it is possible that wrist-worn sensors indirectly capture motion of other joints and segments as well and that this information is represented in the selected features we used to estimate the ARAT scores. However, this statement remains speculative and further research would be required to systematically investigate how the number and placement of the sensor units, as well as the direct and indirect measurement of movements, contribute to the accuracy of clinical scores estimations. This question has never been addressed so far, neither in studies that estimated different clinical scores with larger numbers of sensors (Patel et al., 2010; Adans-Dester et al., 2020), nor in reviews of clinical assessments with wearable sensors (Oña Simbaña et al., 2019; Kim et al., 2021; Boukhennoufa et al., 2022).

Since no previous study estimated ARAT scores from wearable sensors we compare our results to studies that either used different motion sensing techniques to estimate ARAT scores, or studies that used wearable sensors and estimated scores of different clinical tests of ICF activity capacity. For these studies, we inspected coefficients of determination for the relationship between clinical and estimated total scores and (when reported) the estimation error for the difference between clinical and estimated total scores. The results of our study fall in the range of previously achieved results. Alt Murphy et al. (2012) predicted total ARAT scores using kinematic data from marker-based motion capture and observed moderately strong association between clinical and estimated total scores (*R* ^2^ = 0.67). Patients performed a single 3D reaching task and a pre-selected set of movement features were calculated. Kinematic features included: smoothness of the arm endpoint, total movement time, trunk displacement and peak angular velocity of the elbow. The ARAT scores of the patients were obtained in a separate session, then a regression model predicted the total ARAT scores from the kinematic metrics. Olesh et al. (Olesh et al., 2014) estimated scores of the FMA-UE using kinematic data from a low-cost depth sensing camera. Clinical and estimated total FMA-UE scores showed strong association and small estimation errors (*R* ^2^ = 0.86, RMSE = 7.7%). The FMA-UE is a clinical test of ICF function capacity and is intended to assesses more fine-grained movements than the ARAT, but the scale was applied to a subset of movement tasks of the FMA-UE and the ARAT gross movement domain, which makes these results comparable to ours.

Other studies used wearable sensors but estimated different clinical test scales at the ICF activity capacity level. Previous studies estimated the Functional Ability Scale (FAS, which is a subscale of the Wolf Motor Function Test) based on data collected during the execution of a subset of the FAS tasks (Patel et al., 2010; Sapienza et al., 2017; Adans-Dester et al., 2020), using two (on wrist and sternum) or six sensors (distributed over fingers, forearm, upper arm and sternum). *R* ^2^ ranged from 0.79 to 0.97 and RMSE from 2.9% to 7.6%. Other studies estimated the Chedoke Arm and Hand Activity Inventory (CAHAI) based on data collected in free-living settings with two wrist worn sensors (Chen et al., 2020; Tang et al., 2020), with *R* ^2^ ranging from 0.61 to 0.92, and RMSE from 3.1% to 12.0%. Compared to these results, our approach falls in the same range with the advantage of using just two wrist worn sensors.

One strength of this study is the minimalistic sensor setup, which minimizes costs, setup time and device obtrusion, all of which are barriers to the wide spread use of kinematic assessments of motor functioning (Saes et al., 2022). The hardware costs of commercially available inertial sensors, approximately $50 per sensor unit, are relatively low as compared to those of optoelectrical camera systems, approximately $10’000 per system, which are the current gold-standard for clinical motion analysis. Additionally, the same set up is frequently used to measure other aspects of motor functioning (Oña Simbaña et al., 2019; Kim et al., 2021; Boukhennoufa et al., 2022). For example, many studies collected data during activities of daily living or free-living settings and aimed to develop new measures of ICF activity performance, such as quantifications of impaired arm use (e.g., (Bailey et al., 2015; Lee et al., 2019)). Hence, this setup and our analysis have great potential to be applied across the entire continuum of care. It is also worth pointing out that we only used statistical features of acceleration and angular velocity data, in time and frequency domain. These features are easy to obtain from most wearable inertial movement sensors. Hence, the approach is easier to apply and is less biased than solutions that require pre-selection and computation of kinematic features, such as the smoothness of arm endpoint movements or specific joint angles (e.g., Olesh et al., 2014; Kim et al., 2016).

The current study has several limitations. A first limitation is the small sample size. A larger and more diverse sample might increase the prediction accuracy and robustness of the model. In addition, we only included persons with stroke, and it could be interesting to include patients with other neurological disorders as well to further explore the applicability of the sensor-based ARAT estimations. Second, since only one evaluator per participant conducted the ratings we can only assume that the variability between evaluators had only a minor effect on the rating results. Third, other drawbacks are inherent to the use of clinical scores as reference information for training a machine learning model, and the fact that such a model only reproduces the information represented in the clinical scores. Hence, the information contained in the estimated scores depends on that contained in the clinical scores. We assume that the ARAT contains information about movement quality, similar to clinical studies about the ARAT (Yozbatiran et al., 2008), and similar to previous studies which used the FAS to capture information about movement quality (Sapienza et al., 2017; Adans-Dester et al., 2020). These scales, however, assign a task score based on a combination of criteria, some of which might be associated with movement quality only indirectly (Demers and Levin, 2017).

Finally, even though estimated ARAT scores provide an objective and easily interpretable quantification of movement quality, they share the same discrete scale as the underlying, subjective clinical score. Clinical scores are embedded in the field so much that every new method that can estimate previously established clinical scores starts with a clear advantage. Still, scientific research should not stop here. It is worth to reiterate that the estimation of clinical scores is just one way to quantify movement quality using wearable sensors, and that this effort should be complemented with kinematic measures, since these provide quantification of movement quality on a continuous scale (e.g., Schwarz et al., 2019; Formstone et al., 2021). However, while wearable inertial sensor data were already used to explore kinematic measures of movement quality (Repnik et al., 2018), the selection and clinical validation of useful measures is still outstanding.

## 5 Conclusion

The present study demonstrates that it is possible to estimate ARAT task and sum scores with sufficient accuracy for clinical applications using wearable inertial sensors. More specifically, estimation errors smaller than the detectable and important changes of the observation-based ARAT were obtained. The proposed method uses a minimal sensor setup of only one sensor per evaluated arm, which offers a simple, objective, fast and inexpensive way to assess the quality of upper extremity motor functioning across clinical and remote settings. Hence, the current study is opening the doors to more objective and potentially unsupervised assessments of arm and hand motor functioning, in particular at the ICF activity capacity level.

## Data Availability

The raw data supporting the conclusion of this article will be made available by the authors, without undue reservation.
